# Dr. Clapp’s Reply

**Published:** 1900-12

**Authors:** 


### DR. CLAPP'S REPLY.

Upon another page of this number will be found Dr. Clapp's reply to an editorial in the September issue of this journal. The pages of the International Dental Journal are always open to the courteous discussion of all matters of general interest to dentistry. It was established that the dental profession should have a journal for the expression of all shades of opinion untrammelled by commercial or private interests.

It is thought, however, that Dr. Clapp has failed to catch the drift of the editorial he attempts to criticise. The editor made no effort to narrow his observation to any particular State examining board, but endeavored to show that a general weakness existed in the entire plan of regulating dental practice by the methods adopted. The State board of which Dr. Clapp is an honored member may be all that he claims for it. Whether this be true or not does not, in the slightest degree, invalidate the expressed opinion that the entire system is wrong in principle and worse in practice. It would, therefore, have been more gratifying had Dr. Clapp taken up the whole question and given us solid arguments to controvert the growing sentiment that State examining boards, to be of any value, must be organized upon an altogether different basis from that at present in force. The results are not satisfactory, and, as a whole, are discreditable to an intelligent profession.

